# Assessing the link of malnutrition with diabetes and mortality risk in heart failure patients

**DOI:** 10.1002/ehf2.15263

**Published:** 2025-03-12

**Authors:** Konstantinos Prokopidis, Yang Chen, Yang Liu, Ziyi Zhong, Jordi Morwani‐Mangnani, Daniel J. Cuthbertson, Rajiv Sankaranarayanan, Gregory Y.H. Lip, Masoud Isanejad

**Affiliations:** ^1^ Department of Musculoskeletal Ageing and Science, Institute of Life Course and Medical Sciences University of Liverpool Liverpool UK; ^2^ Liverpool Centre for Cardiovascular Science at University of Liverpool Liverpool John Moores University and Liverpool Heart & Chest Hospital Liverpool UK; ^3^ Department of Cardiovascular and Metabolic Medicine, Institute of Life Course and Medical Sciences University of Liverpool Liverpool UK; ^4^ Department of Cardiovascular Medicine, The Second Affiliated Hospital, Jiangxi Medical College Nanchang University Nanchang Jiangxi China; ^5^ Department of Biomedical Data Sciences, Section of Molecular Epidemiology Leiden University Medical Center Leiden The Netherlands; ^6^ Liverpool University Hospitals NHS Foundation Trust Liverpool UK; ^7^ Department of Clinical Medicine, Danish Center for Health Services Research Aalborg University Aalborg Denmark

**Keywords:** Controlling nutritional status, Diabetes, Geriatric nutritional risk index, Heart failure, Malnutrition, Mortality

## Abstract

**Aims:**

Malnutrition is increasingly recognized as a significant factor influencing the clinical outcomes of patients with heart failure (HF). Diabetes exacerbates risks like hospitalizations and mortality due to cardiovascular complications. The aim of this study was to explore the association of malnutrition with diabetes and its prognostic impact on all‐cause and cardiovascular mortality in patients with HF, using the nutritional assessment tools, controlling nutritional status (CONUT) score and geriatric nutritional risk index (GNRI).

**Methods and results:**

Data were obtained from the National Health and Nutrition Examination Survey (1999–2018). Malnutrition was assessed using the CONUT score and GNRI. Multivariate logistic regression models were used to assess the association between malnutrition and diabetes. And Cox proportional hazards models were used to assess the risk of death from malnutrition combined with or without diabetes in HF separately. In addition, propensity score matching and inverse probability weighting were used to adjust for confounders for logistic regression model and Cox proportional hazards model analyses. Finally, subgroup analyses were performed. This study included 1501 HF participants (median age 70.0 years; 43.8% females), including 586 (39.0%) with diabetes. Continuous CONUT was significantly associated with diabetes in HF (OR 1.19, 95% CI: 1.08–1.32, *P* < 0.001) and remained significant after propensity score matching and inverse probability weighting. Similar relationships exist for categorized CONUT, but GNRI was not associated with diabetes in HF. Then, 1500 participants completed follow‐up (5.6 [2.8–9.7] years). Elevated continuous CONUT was related to higher all‐cause (HR = 1.18, 95% CI: 1.09–1.29, *P* < 0.001) and cardiovascular mortality (HR = 1.26, 95% CI: 1.12–1.42, *P* < 0.001) in HF patients with diabetes. And increased continuous CONUT was linked to higher all‐cause (HR = 1.12, 95% CI: 1.03–1.22, *P* < 0.001) and cardiovascular mortality (HR = 1.20, 95% CI: 1.07–1.35, *P* < 0.001) in HF patients without diabetes. Similar relationships exist for categorized CONUT.

**Conclusions:**

Malnutrition assessed by CONUT is linked to higher diabetes prevalence in HF, influenced by altered albumin, cholesterol and lymphocyte levels. CONUT also predicts all‐cause and cardiovascular mortality in HF with and without diabetes. Future research should consider dietary assessments and body composition analysis to assess malnutrition in HF patients.

## Introduction

Heart failure (HF) is a cardiovascular syndrome accompanied by structural and/or functional abnormalities of the heart, resulting in higher intracardiac pressure and inadequate cardiac output leading to classical clinical symptoms. HF is classified into distinct phenotypes based on left ventricular ejection fraction (LVEF): HF with reduced ejection fraction (HFrEF; LVEF ≤40%), HF with mid‐range ejection fraction (HFmrEF; LVEF 41%–49%), and HF with preserved ejection fraction (HFpEF; LVEF ≥50%).^1^ Although these phenotypes share common clinical features, their underlying pathophysiology and risk factors differ. HFrEF relates structurally to cardiac ischaemia, characterized by increased susceptibility to eccentric cardiac hypertrophy, while HFpEF is observed with cardiometabolic comorbidities such as obesity, hypertension and type 2 diabetes, structurally linked to greater concentric hypertrophy.^2^

A mediating factor of heart failure severity and/or incidence may be reflected in malnutrition due to under or overnutrition.^3^ Undernutrition may lead to energy and nutrient deficiency, impairing cardiac muscle function and exacerbating HF symptoms. A prominent feature of undernutrition in HF is cardiac cachexia, which is characterized primarily by rapid involuntary weight loss.^4^ However, the term ‘malnutrition’ that is often used in cachexia research, should be avoided as it implies that the condition is primarily driven by nutritional deficiencies and can be corrected through adequate nutrition or by addressing issues with nutrient absorption or utilization. Conversely, overnutrition can contribute to obesity, dyslipidaemia and hypertension, all of which are major risk factors for HF.^5^ The prevalence of malnutrition in HF is challenging to ascertain due to the absence of standardized diagnostic methods, that can be influenced by the screening tools used and the characteristics of the studied population, leading to a varying prevalence of 2.7 to 90%.^6^ Some validated tools used for the assessment of malnutrition in HF are the Controlling Nutritional Status (CONUT) score and the Geriatric Nutritional Risk Index (GNRI).^7^ Mild to severe malnutrition based on CONUT is linked to a significantly higher mortality risk in HF.^8^ Based on the National Health and Nutrition Examination Survey (NHANES), energy intake may, in part, influence all‐cause mortality in HF,^9^, ^10^ while additional increases in protein (primarily from red meat) and vegetable consumption may improve nutritional status.^11^ Recognizing patients with HF and malnutrition by healthcare professionals could promote improved risk stratification and may potentially improve prognosis through individualized nutritional support.

Moreover, a worsened malnutrition profile is linked to altered glycemic and lipoprotein levels that are associated with multiple cardiovascular risk factors, including diabetes, a major contributor to HF risk.^12^ More specifically, epidemiologically, the prevalence of type 2 diabetes (T2D) in HF ranges between 20% and 40%.^13^ T2D may exacerbate hospitalizations,^14^ mortality^15^ or both combined^16^ in HF due to higher incidence of atherosclerosis, hypertension, microvascular complications and/or kidney dysfunction. Considering this and the importance of well‐nourishment in HF, in this study, we aim to investigate whether diabetes is linked to a higher risk of malnutrition in patients with HF using the CONUT and GNRI scores. As a secondary objective, we will attempt to explore the prognostic impact of malnutrition in HF with versus without diabetes. These objectives could help identify key clinical indicators or biomarkers observed in HF patients with diabetes at a higher risk of malnutrition that may guide targeted interventions to improve clinical outcomes in this population. Lastly, considering the heterogeneity of malnutrition assessment tools due to their different components, it could provide insights on their individual predictive and prognostic value.

## Methods

### Study design and subjects

Data from study participants (*n* = 101 316) were collected from consecutive survey cycles in NHANES from 1999 to 2018. From these cycles, all participants with available data regarding heart failure and diabetes were subsequently collected (*n* = 1906). A written informed consent from all participants and approval has been obtained from the Ethics Review Board of the National Center for Health Statistics and this study was conducted in accordance with the principles of the Declaration of Helsinki.

### Malnutrition assessments

The Controlling Nutritional Status (CONUT) score is a nutritional screening tool that evaluates three key parameters: serum albumin concentration, total lymphocyte count and total cholesterol concentration.^17^ We classified our groups into normal nutrition (0–1 CONUT score) and malnutrition (2–12 CONUT score).

The Geriatric Nutritional Risk Index (GNRI) is a nutritional assessment tool, evaluating serum albumin levels and weight changes to estimate nutritional risk.^18^ In this study, we classified our groups into quartiles (Q1: GNRI ≤ 109.95, Q2: 109.95 < GNRI ≤ 118.67, Q3: 118.67 < GNRI ≤ 128.53, Q4: 128.53 > GNRI).

The parameters used to estimate these two tools are presented in Method S1.

### Covariates definitions

Age (years), sex, race, body mass index (BMI) (kg/m^2^), levels of education, poverty to income ratio, smoking status, alcohol consumption, abdominal obesity, arthritis, coronary heart disease, angina, heart attack, stroke, liver disease, cancer and malignancy, hypertension, use of anti‐hypertensive or cholesterol‐lowering medications, energy intake (g/day), protein intake (g/day), carbohydrate intake (g/day), fat intake (g/day), fibre intake (g/day), serum glucose (mg/dL), serum iron (mcg/dL), serum creatinine (mg/dL) and estimated glomerular filtration rate (eGFR) (mL/min/1.73 m^2^) were used as covariates.

### Statistical analysis

Continuous variables were expressed as mean ± standard deviation (SD), while the categorized variables were presented as counts (percentages). Restricted cubic splines (RCS) were used to evaluate the associations between malnutrition scores and diabetes in HF. Multivariate logistic regression models were used to assess the associations of malnutrition scores with diabetes in HF using odds ratios (OR) and 95% confidence intervals (CI). The association of malnutrition scores with all‐cause or cardiovascular mortality was assessed by multivariate Cox proportional hazards models with hazard ratios (HR) and 95% CI. In terms of covariate adjustment, an initial multicollinearity test was performed, and the covariates without strong multicollinearity (the variance inflation factor ≤10) were included as confounders. Model 1 was the unadjusted model, in Model 2 our models were adjusted by age, sex, race, education and PIR, in Model 3 by nutrients (protein intake and fibre intake), BMI, abdominal obesity, smoking and alcohol consumption, and in Model 4 by comorbidities (arthritis, coronary heart disease, angina, heart attack, stroke, liver disease, cancer and malignancy and hypertension), medications (hypertension medication and cholesterol medication), and laboratory measurements (serum glucose, iron, creatinine and eGFR). In addition, Kaplan–Meier curve analysis was used to assess the relationship between different levels of malnutrition scores and all‐cause or cardiovascular mortality.

Furthermore, when CONUT or GNRI was found to be significantly associated with diabetes in HF, in order to reduce the effect of confounders, we used the propensity score matching (PSM) method and inverse probability weighting (IPTW) to balance baseline characteristics between the high CONUT/GNRI and low CONUT/GNRI groups. For PSM, we used Logistic regression models adjusted by Model 4 to estimate propensity scores for each subject in the high CONUT/GNRI group. After estimating the propensity scores, we matched between the treatment and control groups using 1:1 nearest‐neighbour matching. For IPTW, we calculated inverse probability weights for each subject. In the weighted sample, we examined the balance between high CONUT/GNRI and low CONUT/GNRI on baseline characteristics, using standardized differences as an assessment metric to ensure that all variables were balanced after weighting. Subsequently, logistic regression or Cox proportional risk models were performed to assess the association between CONUT or GNRI and diabetes in HF, respectively.

Subgroup analyses employing multivariate logistic regression for continuous or categorized CONUT and GNRI scores were based on age (<70 vs. ≥70 years), sex (males vs. females), BMI (<30 vs. ≥30 kg/m^2^), presence of abdominal obesity, arthritis, coronary heart disease, heart attack, cancer and malignancy, hypertension and stroke. Also, for subgroup analyses of mortality risk, we performed multifactorial Cox proportional hazards models for continuous or categorized CONUT scores based on diabetes and non‐diabetes. All data analyses were performed using packages (‘survival’, ‘survminer’, ‘dplyr’, ‘ggplot2’, ‘gtsummary’, ‘haven’ and ‘rcssci’) in R software (version 4.3.2). A *P* value <0.05 was used to determine significance.

## Results

### Baseline characteristics

**Figure 1:**
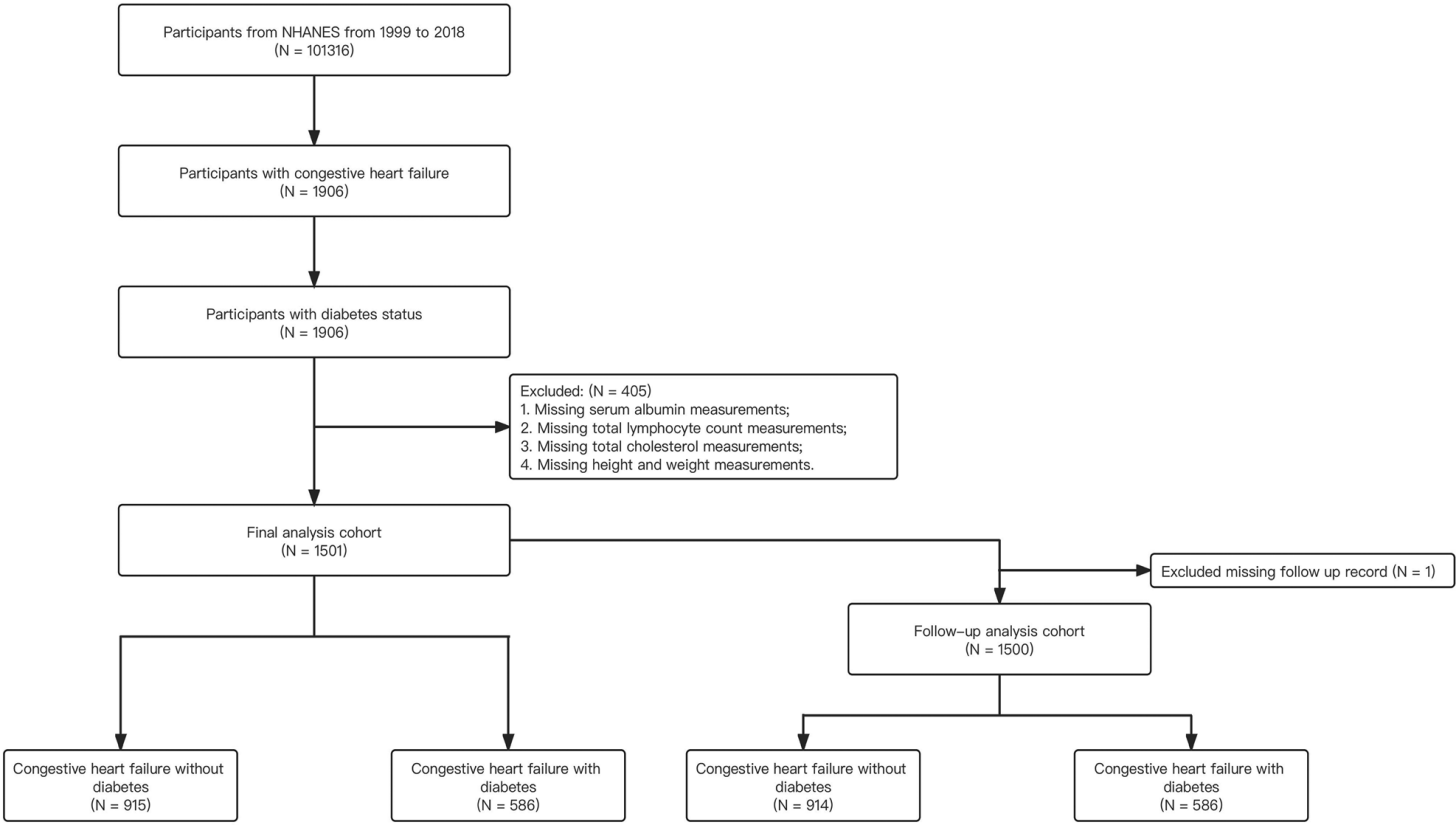
Flowchart of this study. NHANES, The National Health and Nutrition Examination Survey.

This study included 1501 participants (*Figure* *1*). Baseline characteristics of sociodemographic, anthropometric, nutritional and comorbidity status among all participants are outlined (*Table* *1*). Of the 1906 participants, 405 were excluded due to missing serum albumin, total lymphocyte count, total cholesterol, and height and weight measurements. Considering one participant had a missing follow‐up record, 1500 participants were enrolled in the present study (HF without diabetes [*n* = 914]; HF with diabetes [*n* = 586]). The cohort had a median age of 70.0 (interquartile range [IQR] 60.0–79.0), consisted of 43.8% females. Patients with HF and diabetes had a significantly higher BMI (32.3 [28.3–38.4] kg/m^2^) compared with those without diabetes (28.4 [25.1–33.4] kg/m^2^) (*P* < 0.01). This difference also corresponded with increased prevalence of abdominal obesity (with diabetes: 82.9% vs. without diabetes: 67.5%; *P* < 0.01). CONUT scores were identical between the two groups (1.0 [0.0–2.0]), while patients with diabetes exhibited higher GNRI (122.3 [112.6–131.8]) versus those without diabetes (116.3 [108.6–125.9]).

**Table 1:** Baseline characteristics and outcomes of patients with heart failure

Characteristic	All (*N* = 1501)	Without diabetes (*N* = 915)	With diabetes (*N* = 586)	*P*
Age, years	70.0 (60.0–79.0)	70.0 (59.0–80.0)	69.0 (62.0, 77.0)	0.391
Age, *n* (%)				0.204
<70 years	722 (48.1)	428 (46.8)	294 (50.2)	
≥70 years	779 (51.9)	487 (53.2)	292 (49.8)	
Female, *n* (%)	657 (43.8)	390 (42.6)	267 (45.6)	0.263
Race, *n* (%)				<0.001
Mexican American	162 (10.8)	86 (9.4)	76 (13.0)	
Other Hispanic	102 (6.8)	58 (6.3)	44 (7.5)	
Non‐Hispanic White	820 (54.6)	542 (59.2)	278 (47.4)	
Non‐Hispanic Black	344 (22.9)	184 (20.1)	160 (27.3)	
Others	73 (4.9)	45 (4.9)	28 (4.8)	
BMI, kg/m^2^	30.1 (26.0–35.6)	28.4 (25.1–33.4)	32.3 (28.3–38.4)	<0.001
BMI, *n* (%)				<0.001
<28 kg/m^2^	292 (19.5)	226 (24.7)	66 (11.3)	
28–30 kg/m^2^	448 (29.8)	312 (34.1)	136 (23.2)	
>30 kg/m^2^	761 (50.7)	377 (41.2)	384 (65.5)	
Abdominal obesity, *n* (%)	1104 (73.6)	618 (67.5)	486 (82.9)	<0.001
Education, *n* (%)				0.040
Less than high school	593 (39.5)	339 (37.0)	254 (43.3)	
High school	367 (24.5)	238 (26.0)	129 (22.0)	
More than high school	541 (36.0)	338 (36.9)	203 (34.6)	
Poverty‐income ratio, *n* (%)				0.455
PIR < 1.0	337 (22.5)	199 (21.7)	138 (23.5)	
1.0 ≤ PIR < 2.0	507 (33.8)	304 (33.2)	203 (34.6)	
PIR ≥ 2.0	657 (43.8)	412 (45.0)	245 (41.8)	
Smoking, *n* (%)				0.017
Never	615 (41.0)	363 (39.7)	252 (43.0)	
Quit	604 (40.2)	359 (39.2)	245 (41.8)	
Now	282 (18.8)	193 (21.1)	89 (15.2)	
Annual alcohol intake (over 12 units), *n* (%)	873 (58.2)	548 (59.9)	325 (55.5)	0.096
Comorbidity, *n* (%)				
Arthritis	895 (59.6)	515 (56.3)	380 (64.8)	0.001
Coronary heart disease	633 (42.2)	363 (39.7)	270 (46.1)	0.016
Angina	392 (26.1)	224 (24.5)	168 (28.7)	0.081
Heart attack	667 (44.4)	391 (42.7)	276 (47.1)	0.099
Stroke	302 (20.1)	166 (18.1)	136 (23.2)	0.018
Liver condition	111 (7.4)	66 (7.2)	45 (7.7)	0.762
Cancer and malignancy	311 (20.7)	195 (21.3)	116 (19.8)	0.514
Hypertension	1168 (77.8)	670 (73.2)	498 (85.0)	<0.001
Medications, *n* (%)				
Hypertension	1347 (89.7)	793 (86.7)	554 (36.9)	<0.001
Cholesterol	1102 (73.4)	612 (66.9)	490 (83.6)	<0.001
Nutrient intake (daily)				
Energy, kcal	1628.0 (1220.0–2118.0)	1698.5 (1282.0–2214.0)	1545.0 (1155.6–1974.1)	<0.001
Protein, g	63.6 (47.7–84.6)	64.3 (48.2–85.9)	62.6 (46.3–81.3)	<0.001
Carbohydrate, g	199.5 (148.0–262.3)	211.8 (154.1–276.5)	183.9 (136.3–245.3)	<0.001
Fat, g	61.7 (42.4–86.0)	63.5 (43.9–89.9)	59.1 (40.8–80.3)	0.015
Fibre, g	12.9 (8.8–18.5)	13.0 (8.7–18.3)	12.8 (8.9–18.8)	0.987
Laboratory measurements				
Serum glucose, mg/dL	102.0 (92.0–128.0)	96.0 (89.0–106.0)	129.0 (103.0–180.0)	<0.001
Serum iron, μmol/L	72.0 (54.0–93.0)	75.0 (56.0–97.0)	67.0 (52.0–87.0)	<0.001
Serum creatinine, μmol/L	1.0 (0.9–1.4)	1.0 (0.8–1.3)	1.1 (0.9–1.5)	<0.001
HbA1c, %(mmol/mol)	5.90 (41) (5.50–6.60) (37–49)	5.60 (38) (5.40–6.00) (36–42)	6.80 (51) (6.20–7.73) (44–61)	<0.001
eGFR, mL/min/1.73 m^2^	74.75 (55.90–96.35)	77.84 (59.10–99.19)	69.07 (49.03–88.61)	<0.001
CONUT	1.0 (0.0–2.0)	1.0 (0.0–2.0)	1.0 (0.0–2.0)	<0.001
0–1	938 (62.5%)	600 (65.57%)	338 (57.68%)	
≥2	563 (37.5%)	315 (34.43%)	248 (42.32%)	
GNRI	118.7 (110.0–128.5)	116.3 (108.6–125.9)	122.3 (112.6–131.8)	<0.001
<98	57 (3.8%)	44 (4.81%)	13 (2.22%)	
≥98	1444 (96.2%)	871 (95.19%)	573 (97.78%)	
Outcomes, *n* (%)^a^				
All‐cause mortality	786 (52.4)	465 (50.9)	321 (54.8)	0.153
Cardiovascular mortality	305 (20.3)	176 (19.3)	129 (22.0)	0.212

BMI, body mass index; CONUT, controlling nutritional status; eGFR; estimated glomerular filtration rate; GNRI, geriatric nutrition risk index; HF, heart failure; PIR, poverty income ratio.

^a^ One HF patient was lost to follow‐up, *N* = 1500 for the outcome section.

### Association of CONUT and GNRI with diabetes in HF

**Figure 2:**
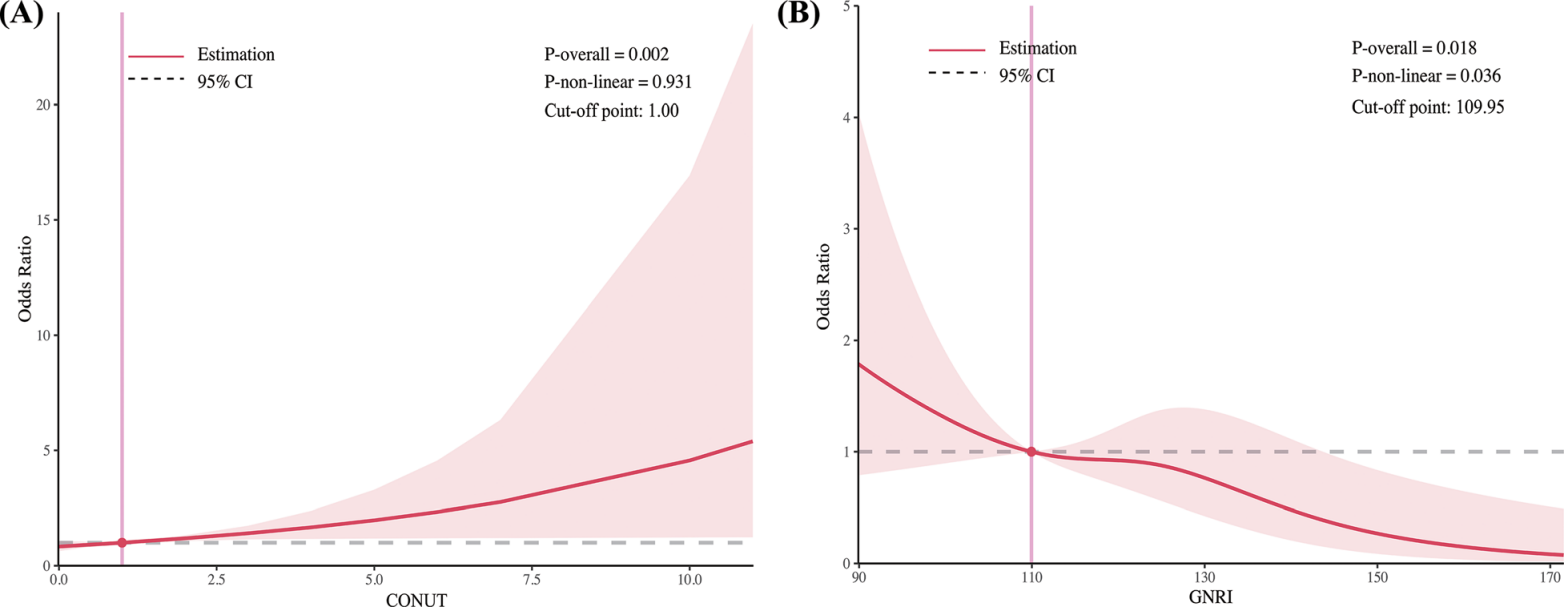
Restricted cubic splines analyses between CONUT (A)/GNRI (B) with diabetes in heart failure. CONUT, controlling nutritional status; GNRI, geriatric nutritional risk index.

For the restricted cubic spline analyses, *Figure* *2* *A* shows that the CONUT is significantly linked to diabetes in HF (*P* overall = 0.002), but no non‐linear relationship (*P* non‐linear = 0.931). On the other hand, GNRI was associated with diabetes in HF (*P* overall = 0.018), with a nonlinear association (*P* non‐linear = 0.036).

*Table* *2* shows the results of multivariate logistic regression regarding the association of CONUT or GNRI with diabetes in HF. For the GNRI, continuous GNRI was associated with diabetes in HF (OR 0.97, 95% CI: 0.95–0.99, *P* = 0.002), but categorized GNRI was not. Higher CONUT as a continuous variable was linked to increased prevalence of diabetes in HF (OR 1.19, 95% CI: 1.08–1.32, *P* < 0.001). Furthermore, no significant interactions were observed in continuous nor categorized GNRI scores (Table S2).

**Table 2:** Multivariate logistic regression analyses for continuous/categorized CONUT/GNRI and diabetes in HF patients

	Model 1			Model 2			Model 3			Model 4		
	OR	95% CI	*P*	OR	95% CI	*P*	OR	95% CI	*P*	OR	95% CI	*P*
**CONUT**												
Continuous	1.20	1.11–1.29	<0.001	1.23	1.13–1.33	<0.001	1.26	1.16–1.38	<0.001	1.19	1.08–1.32	<0.001
Categorized												
Score 0–1	Reference			Reference			Reference			Reference		
Score 2–12	1.40	1.13–1.73	0.002	1.48	1.20–1.86	<0.001	1.62	1.28–2.04	<0.001	1.41	1.07–1.86	0.014
**GNRI**												
Continuous	1.03	1.02–1.03	<0.001	1.3	1.02–1.03	<0.001	0.94	0.92–0.96	<0.001	0.97	0.95–0.99	0.002
Categorized												
Q1	Reference			Reference			Reference			Reference		
Q2	1.68	1.23–2.30	0.001	1.74	1.27–2.39	<0.001	1.21	0.86–1.72	0.276	1.31	0.86–1.99	0.204
Q3	2.34	1.72–3.19	<0.001	2.48	1.81–3.41	<0.001	1.19	0.79–1.79	0.396	1.46	0.90–2.38	0.126
Q4	3.14	2.30–4.27	<0.001	3.21	2.33–4.43	<0.001	0.83	0.47–1.45	0.504	1.29	0.67–2.51	0.447

*Note:* GNRI grouping by Q1: GNRI ≤ 109.95, Q2: 109.95 < GNRI ≤ 118.67, Q3: 118.67 < GNRI ≤ 128.53, Q4: 128.53 < GNRI. Model 1: Unadjusted. Model 2: Model 1 adjusted by age, sex, race, education and PIR. Model 3: Model 2 adjusted by nutrients (protein and fibre intake), BMI, abdominal obesity, smoking and drinking. Model 4: Model 3 adjusted by comorbidities (arthritis, coronary heart disease, angina, heart attack, stroke, liver condition, cancer and malignancy and hypertension), medications (hypertension and cholesterol), laboratory measurements (serum glucose, serum iron and serum creatinine).

Abbreviations: BMI, body mass index; CI, confidence interval; CONUT, controlling nutritional status; GNRI, geriatric nutrition risk index; HF, heart failure; OR, odds ratio; PIR, poverty income ratio.

**Figure 3:**
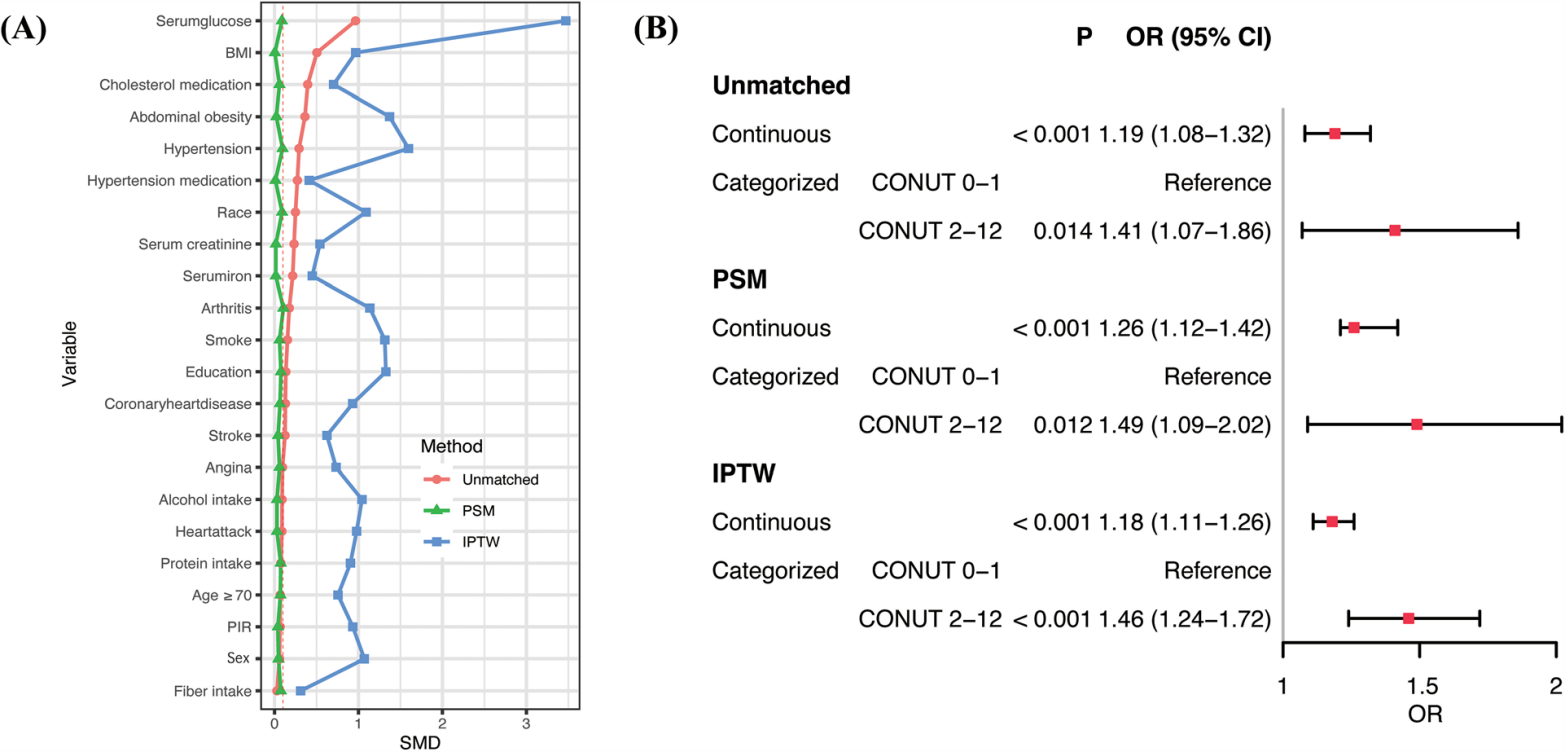
PSM and IPTW for CONUT with diabetes in heart failure. (A) Visualization of the PSM and IPTW matching and equalization. (B) Association between CONUT and diabetes in HF by using unmatched logistic regression model, and logistic regression model after PSM and IPTW. BMI, body mass index; CI, confidence interval; CONUT, controlling nutritional status; IPTW, inverse probability of treatment weighting; OR, odds ratio; PIR, poverty to income ratio; PSM, propensity score matching.

Compared with a lower CONUT (<2), a higher score (≥2) was related to higher risk of diabetes in HF (OR 1.41, 95% CI: 1.07–1.86, *P* = 0.014). In addition, after PSM and IPTW (*Figure* *3*), CONUT remained significantly associated with diabetes in HF (PSM: continuous CONUT, OR 1.26, 95% CI: 1.12–1.42, *P* < 0.001; categorized CONUT [≥2 compared with <2], OR 1.49, 95% CI: 1.09–2.02, *P* = 0.012) (IPTW: continuous CONUT, OR 1.18, 95% CI: 1.11–1.26, *P* < 0.001; categorized CONUT [≥2 compared with <2], OR 1.46, 95% CI: 1.24–1.72, *P* < 0.001). In addition, no significant interaction was observed in categorized CONUT scores, but there was an interaction of arthritis and cancer/malignancy for continuous CONUT and diabetes in HF (*Table* *3*).

**Table 3:** Subgroup analyses of multivariate logistic regression for continuous/categorized CONUT and diabetes in HF patients

		Categorized CONUT				Continuous CONUT			
Subgroups		OR	95% CI	*P*	*P* for interaction	OR	95% CI	*P*	*P* for interaction
Age ≥ 70 years	Yes	1.52	1.05–2.21	0.027	0.757	1.19	1.02–1.39	0.03	0.892
	No	1.34	0.86–2.06	0.193		1.20	1.05–1.37	0.007	
Sex	Female	1.39	0.90–2.16	0.138	0.836	1.16	1.00–1.36	0.055	0.864
	Male	1.48	1.03–2.12	0.034		1.22	1.07–1.39	0.003	
BMI ≥ 30 kg/m^2^	Yes	1.64	1.12–2.39	0.010	0.145	1.11	0.95–1.28	0.171	0.151
	No	1.15	0.75–1.75	0.524		1.27	1.11–1.46	<0.001	
Abdominal obesity	Yes	1.56	1.14–2.14	0.006	0.925	1.23	1.09–1.38	<0.001	0.309
	No	1.15	0.66–2.03	0.620		1.12	0.93–1.34	0.237	
Arthritis	Yes	1.16	0.81–1.66	0.405	0.053	1.10	0.97–1.25	0.140	0.038
	No	2.09	1.32–3.33	0.002		1.37	1.16–1.62	<0.001	
Coronary heart disease	Yes	1.15	0.73–1.81	0.538	0.591	1.16	0.98–1.37	0.083	0.983
	No	1.58	1.09–2.29	0.015		1.21	1.06–1.38	0.004	
Heart attack	Yes	1.29	0.83–2.03	0.260	0.634	1.21	1.03–1.42	0.024	0.768
	No	1.67	1.16–2.42	0.006		1.21	1.06–1.38	0.004	
Cancer and malignancy	Yes	1.25	0.66–2.38	0.491	0.213	0.96	0.77–1.21	0.757	0.017
	No	1.54	1.12–2.11	0.007		1.26	1.13–1.41	<0.001	
Hypertension	Yes	1.49	1.10–2.04	0.011	0.370	1.2	1.08–1.33	0.001	0.729
	No	1.07	0.52–2.18	0.857		1.13	0.86–1.48	0.390	
Stroke	Yes	0.99	0.52–1.91	0.983	0.139	1.03	0.80–1.32	0.808	0.093
	No	1.57	1.15–2.16	0.005		1.24	1.11–1.38	<0.001	

*Note:* Multivariate logistic regression model adjusted for age, sex, race, education, PIR, nutrient intake (protein intake and fibre intake), BMI, abdominal obesity, smoking, drinking, comorbidities (arthritis, coronary heart disease, angina, heart attack, stroke, liver condition, cancer and malignancy and hypertension), medications (hypertension and cholesterol), laboratory measurements (serum glucose, serum iron and serum creatinine).

Abbreviations: BMI, body mass index; CI, confidence interval; CONUT, controlling nutritional status; HF, heart failure; OR, odds ratio; PIR, poverty income ratio.

### Association of CONUT with mortality in HF with diabetes versus without diabetes

**Figure 4:**
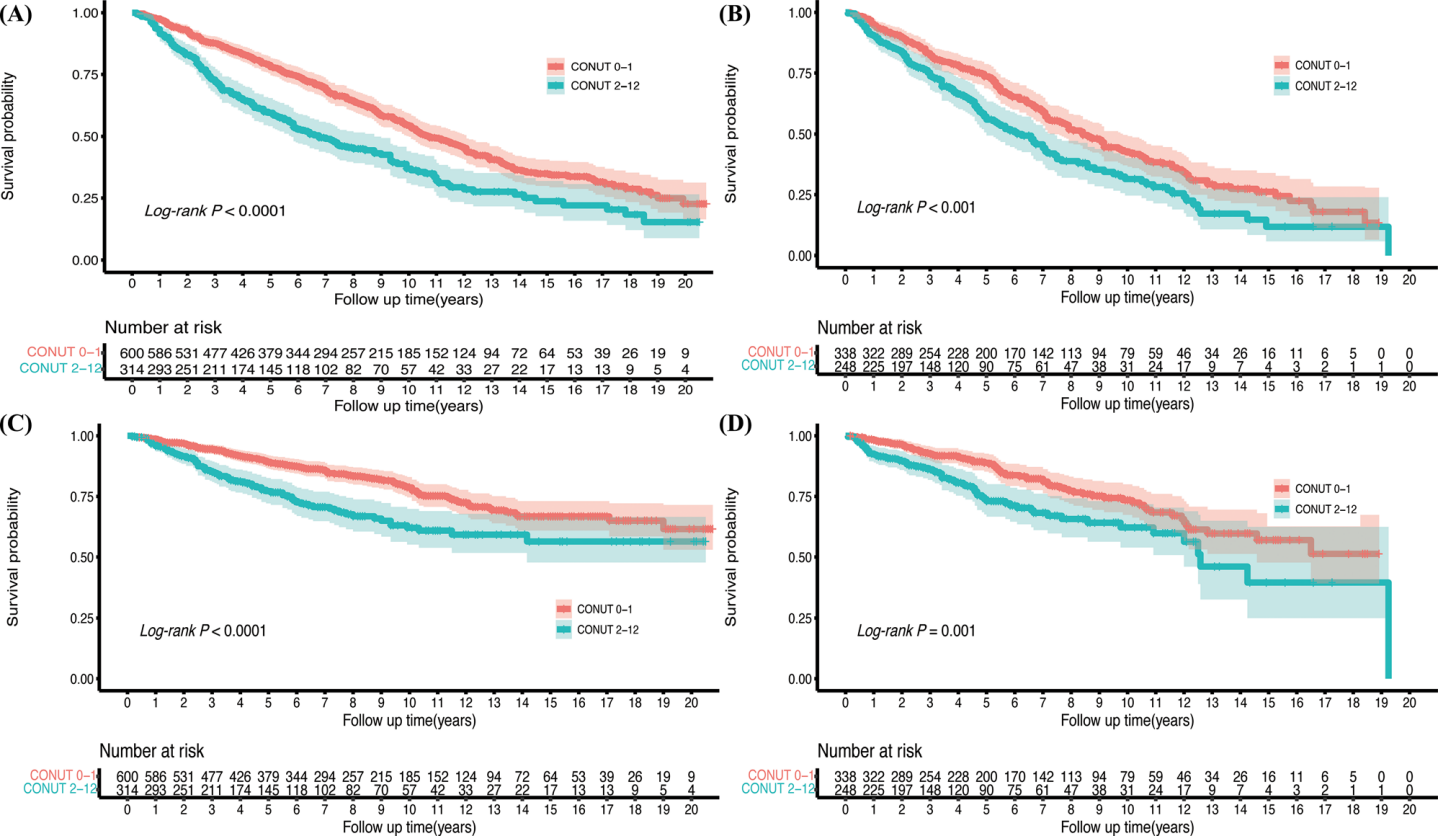
Kaplan–Meier curves for all‐cause mortality and cardiovascular mortality in HF with diabetes and non‐diabetic patients in different groups of CONUT scores. All‐cause (A) and cardiovascular mortality (B) in HF with diabetes. All‐cause (C) and cardiovascular mortality (D) in HF without diabetes. CONUT, controlling nutritional status; HF, heart failure.

A total of 1500 people had completed follow‐up record, and the median (IQR) follow‐up was 5.6 (2.8–9.7) years. *Figure* *4* shows that higher CONUT had significantly more cumulative all‐cause and cardiovascular deaths than lower CONUT. Multivariate Cox proportional hazards models demonstrated that continuous CONUT was a risk factor for all‐cause (HR = 1.12, 95% CI: 1.03–1.22, *P* = 0.007) and cardiovascular mortality (HR = 1.20, 95% CI: 1.07–1.35, *P* = 0.002) in HF without diabetes (*Table* *4*). Similar findings were shown in HF with diabetes (all‐cause mortality: HR = 1.18, 95% CI: 1.09–1.29, *P* < 0.001; cardiovascular mortality: HR = 1.26, 95% CI: 1.12–1.42, *P* < 0.001). When data were treated as categorized, compared with lower CONUT, higher CONUT showed higher risk of cardiovascular mortality (HR = 1.37, 95% CI: 1.01–1.86, *P* = 0.042), but revealed marginal effect with higher all‐cause mortality in HF without diabetes (HR = 1.22, *P* = 0.053). In individuals with HF and diabetes, both all‐cause (HR = 1.32, 95% CI: 1.03–1.67, *P* = 0.026) and cardiovascular mortality (HR = 1.62, 95% CI: 1.13–2.34, *P* = 0.009) remained statistically significant.

**Table 4:** Multivariate Cox proportional hazards for CONUT and different causes of deaths in HF with and without diabetes.

	Without diabetes				Diabetes			
	Incidence	HR	95% CI	*P*	Incidence	HR	95% CI	*P*
Continuous CONUT								
All cause death	321/786	1.12	1.03, 1.22	0.007	465/786	1.18	1.09, 1.27	<0.001
Cardiovascular death	138/333	1.20	1.07, 1.35	0.002	195/333	1.26	1.12, 1.42	<0.001
Categorized CONUT								
All cause death	321/786	1.22	(1.00, 1.49)	0.053	465/786	1.32	(1.03, 1.67)	0.026
Cardiovascular death	138/333	1.37	(1.01, 1.86)	0.042	195/333	1.62	(1.13, 2.34)	0.009

*Note:* Multivariate Cox proportional hazards model adjusted for age, sex, race, education, PIR, nutrient intake (protein intake and fibre intake), BMI, abdominal obesity, smoking, drinking, comorbidities (arthritis, coronary heart disease, angina, heart attack, stroke, liver condition, cancer and malignancy and hypertension), medications (hypertension medications and cholesterol medications) and laboratory measurements (serum glucose, serum iron and serum creatinine).

Abbreviations: BMI, body mass index; CONUT, controlling nutritional status; HF, heart failure; PIR, poverty income ratio.

## Discussion

In this study, we evaluated the association of GNRI and CONUT with diabetes in HF, and their prognostic impact in individuals with HF and diabetes versus without diabetes. Our main findings are as follows: (1) CONUT is significantly associated with diabetes in HF, while GNRI is not. (2) CONUT can be used as a risk assessment tool for all‐cause mortality or cardiovascular mortality in HF, regardless of whether diabetes is present.

Despite patients with HF and diabetes having significantly higher rates of abdominal obesity and BMI compared with those without diabetes in this cohort, we did not find clinical differences in regards to all‐cause mortality risk between groups. However, these phenotypic features may be, in part, responsible for the association of CONUT with diabetes. This suggestion may raise questions of reverse causality, considering that CONUT and GNRI do not assess malnutrition risk based on low dietary values rather than altered blood markers, which could be attributed to significantly increased BMI.

The proposition that obesity could drive malnutrition status in patients with HF and diabetes involves several physiological mechanisms, such as skeletal muscle insulin resistance, altered lipoprotein profile and low‐grade inflammation.^19^ In the context of our HF and diabetes cohort, these metabolic disturbances may exacerbate underlying plasma biomarkers of nutritional status, leading to increased CONUT scores. For example, obesity‐associated low‐grade inflammation may reduce serum albumin levels, a parameter that partially mediates CONUT scores, while dyslipidaemia which is also closely linked with obesity (with elevated triglycerides, total cholesterol and low‐density lipoprotein (LDL) levels) may lower CONUT scores in our HF and diabetes groups. These results may explain the identical median values of CONUT scores between the two cohorts. However, it is worth noting that in our adjusted models, BMI and abdominal obesity were used as covariates. In this case, some markers used to estimate CONUT could have contributed disproportionately, particularly those impacted more by other conditions compared with higher BMI and abdominal obesity. In particular, lower albumin levels (<3.0 g/dL) provide 4 CONUT points, that is two‐fold higher than the maximum scores of total lymphocyte count or total cholesterol could provide. In addition, it is worth noting that some patients with diabetes may exhibit lower levels of lean mass,^20^ or reduced kidney function reflected by estimated glomerular filtration rate (eGFR) levels, or both, which are associated with lower albumin values. In the NHANES dataset, we were unable to assess the body composition of our cohort, considering that data is provided in individuals up to 59 years of age. Therefore, it is plausible to assume that reduced renal function as reflected by the significantly lower concentration of eGFR and higher BMI levels in patients with HF and diabetes could have contributed to increased CONUT scores (*Table* *1*), to a greater extent as opposed to total lymphocyte and cholesterol levels.

### Clinical implications and future directions

The aforementioned findings suggest that CONUT and GNRI may not be entirely reliable for assessing malnutrition risk in patients with HF and diabetes. This is due to alterations in phenotypic and biochemical markers, such as BMI, inflammation and renal function, which may not accurately reflect true nutrient profiles. In particular, GNRI primarily relies on serum albumin levels and body weight, which may not accurately reflect malnutrition in patients with HF, given the increased fluid retention and oedema, and altered body composition. In HF, weight fluctuations and hypoalbuminemia may be more relevant to disease severity rather than true nutritional deficiencies, reducing GNRI's effectiveness compared with CONUT, which includes more diverse markers. Alternatively, malnutrition risk assessment may be more appropriate through records of macro‐ and micronutrient intake, combined with the assessment of individual biochemical parameters used in CONUT and/or GNRI. Nevertheless, CONUT was still shown in this study as a reliable composite indicator for differentiating diabetes in HF and predicting the risk of death in patients with HF. Additionally, considering that changes in the parameters constituting CONUT could reflect alterations in immune/inflammatory responses or fluid retention, which are common in HF, it poses a challenge to implement it clinically in this population. Therefore, looking at the bidirectional relationship between malnutrition and diabetes in HF may require a more holistic approach, assessing biochemical, phenotypic and nutrient intake profiling.

### Strengths and limitations

In the present study, we used widely validated and clinically implemented nutritional assessment tools, namely the CONUT and GNRI scores, adding robustness to our findings. These tools ensure consistency and reliability in assessing the nutritional status of patients. Second, our study employed robust statistical methods to determine the prognosis and efficacy of CONUT and GNRI scores in association with diabetes and mortality risk. Using multiple linear and subgroup analyses, we enhanced the validity and reliability of our findings, allowing for a comprehensive examination of the relationship among nutritional status, diabetes and mortality in individuals with HF.

Our study was prone to several limitations. Limited statistical power for sub‐analyses, particularly due to small subgroup sizes, may diminish our ability to detect significant associations or draw meaningful conclusions. Consequently, larger sample sizes may be necessary for validation. Furthermore, our study acknowledges its limited exploration of low GNRI scores due to the baseline high average score, reflecting a generally well‐nourished cohort with fewer metabolic fluctuations compared with CONUT. Moreover, NHANES does not distinguish between type 1 and type 2 diabetes; therefore, whether one may be linked to a greater risk of malnutrition based on CONUT compared with the other could not be observed in this study. Furthermore, we could not account for specific medications commonly used in this patient group, including sodium‐glucose cotransporter‐2 (SGLT2) inhibitors or glucagon‐like peptide‐1 (GLP‐1) receptor agonists, which may confer protection against all‐cause and cardiovascular mortality by improving cardiovascular outcomes and glycaemic profile in diabetes (i.e., improving albumin levels.^21^ Added to this, since 2008, the methods for diagnosing and treating HF have significantly evolved in alignment with updated HF guidelines. As a result, current studies should focus on populations who have received at least three of the four key pharmacotherapy treatments, as well as ICD/CRT‐D devices. Additionally, the absence of HF parameters, such as the New York Heart Association (NYHA) classification and LVEF levels presents a notable gap. Accordingly, we could not conduct subgroup analysis based on different HF phenotypes, given that HFpEF and HFrEF have different anthropometrics, that have subsequently exhibited different CONUT and GNRI results.

## Conclusions

The findings from NHANES suggest that malnutrition assessed by CONUT is significantly associated with higher diabetes prevalence in HF, which may be attributed to factors altering total cholesterol, lymphocyte count and serum albumin. CONUT shows potential as a risk factor for all‐cause or cardiovascular mortality both in HF with and without diabetes. These results highlight the need for more tailored nutritional assessments (i.e., estimating nutrient intake) in patients with HF, particularly those with diabetes. Future research should explore alternative tools or complementary methods, such as detailed dietary assessments and body composition analysis, to more accurately capture malnutrition risk in this population.

## Conflict of interest

DJC has received investigator‐initiated research funding and consultancy funding from Astra Zeneca, Novo Nordisk, Ipsen and Perpsectum. The other authors declare no competing interests.

## Supporting information


**Table S1.** Results of the multi‐collinearity test.
**Table S2.** Subgroup analyses of multivariate logistic regression for GNRI and diabetes in HF patients.

## Data Availability

The original contributions presented in the study are included in the article. Further inquiries can be directed to the corresponding author.
