# Supplementary material for: Assessing the link of malnutrition with diabetes and mortality risk in heart failure patients
Source: ESC Heart Fail. 2025 Mar 12;12(4):3103–13. doi: 10.1002/ehf2.15263 (PMC12287868; doi:10.1002/ehf2.15263)
Supplement: Supplementary file 1 — Table S1. Results of the multi‐collinearity test. Table S2. Subgroup analyses of multivariate logistic regression for GNRI and diabetes in HF patients. [file EHF2-12-3103-s001.docx]

**Supplementary eMethod.** Estimation of CONUT and GNRI scores.

CONUT assigns scores based on the values of these parameters:

Normal Nutritional Status: CONUT score 0-1

Mild Malnutrition: CONUT score 2-4

Moderate Malnutrition: CONUT score 5-8

Severe Malnutrition: CONUT score 9 or higher

These scores were calculated based on:

CONUT Score = Serum Albumin Score + Total Lymphocyte Count Score + Total Cholesterol Score

Serum Albumin Score:

0 points: Albumin ≥ 3.5 g/dL

2 points: 3.0 ≤ Albumin < 3.5 g/dL

4 points: Albumin < 3.0 g/dL

Total Lymphocyte Count Score:

0 points: ≥ 1600 cells/mm³

1 point: 1200-1599 cells/mm³

2 points: 800-1199 cells/mm³

3 points: < 800 cells/mm³

Total Cholesterol Score:

0 points: Cholesterol ≥ 180 mg/dL

1 point: 140-179 mg/dL

2 points: 100-139 mg/dL

3 points: < 100 mg/dL

GNRI was classified as:

Well-Nourished: GNRI above 98

Mildly Malnourished: GNRI 92-98

Moderately Malnourished: GNRI 82-92

Severely Malnourished: GNRI below 82

GNRI scores were calculated based on:

GNRI = (1.489 × Albumin (g/dL)) + (41.7 × (Current Weight / Usual Weight))

**Supplementary Table S1**. Results of the multi-collinearity test.

| Variable | Variance inflation factor |
| --- | --- |
| Age | 1.25 |
| Gender | 1.50 |
| Race | 1.09 |
| BMI | 2.26 |
| Abdominal Obesity | 2.25 |
| Education | 1.13 |
| PIR | 1.10 |
| Smoke | 1.22 |
| Alcohol intake | 1.22 |
| Arthritis | 1.09 |
| Coronary heart disease | 1.32 |
| Angina | 1.21 |
| Heart attack | 1.25 |
| Stroke | 1.03 |
| Liver condition | 1.05 |
| Cancer & malignancy | 1.06 |
| Hypertension | 1.11 |
| Hypertension medications | 1.22 |
| Cholesterol medications | 1.26 |
| Energy intake | 54.35 |
| Protein intake | 4.93 |
| Carbohydrate intake | 16.73 |
| Fat intake | 12.00 |
| Fiber intake | 2.16 |
| Serum glucose | 1.09 |
| Serum iron | 1.09 |
| Serum creatinine | 1.10 |

Abbreviation: BMI, body mass index; PIR, poverty income ratio.

**Supplementary Table S2.** Subgroup analyses of multivariate logistic regression for GNRI and diabetes in HF patients.

|  |  |  | Categorized GNRI | | | | Continuous GNRI | | |  |
| --- | --- | --- | --- | --- | --- | --- | --- | --- | --- | --- |
| Subgroups |  |  | OR | 95%CI | *P* | *P* for interaction | OR | 95%CI | *P* | *P* for interaction |
| Age ≥ 70 years | Yes | Q1 | Reference | |  | 0.683 | 0.99 | 0.95, 1.02 | 0.506 | 0.662 |
|  |  | Q2 | 1.36 | 0.79, 2.36 | 0.273 |  |  |  |  |  |
|  |  | Q3 | 1.89 | 0.97, 3.70 | 0.061 |  |  |  |  |  |
|  |  | Q4 | 1.63 | 0.60, 4.44 | 0.341 |  |  |  |  |  |
|  | No | Q1 | Reference | |  |  | 0.96 | 0.93, 1.00 | 0.058 |  |
|  |  | Q2 | 1.25 | 0.62, 2.54 | 0.539 |  |  |  |  |  |
|  |  | Q3 | 0.93 | 0.42, 2.08 | 0.853 |  |  |  |  |  |
|  |  | Q4 | 1.01 | 0.38, 2.67 | 0.989 |  |  |  |  |  |
|  |  |  |  |  |  |  |  |  |  |  |
| Gender | Male | Q1 | Reference | |  | 0.921 | 0.97 | 0.93, 1.00 | 0.048 | 0.523 |
|  |  | Q2 | 1.41 | 0.80, 2.51 | 0.235 |  |  |  |  |  |
|  |  | Q3 | 1.43 | 0.73, 2.81 | 0.297 |  |  |  |  |  |
|  |  | Q4 | 1.07 | 0.42, 2.74 | 0.880 |  |  |  |  |  |
|  | Female | Q1 | Reference | |  |  | 0.99 | 0.95, 1.03 | 0.734 |  |
|  |  | Q2 | 1.45 | 0.76, 2.80 | 0.267 |  |  |  |  |  |
|  |  | Q3 | 1.87 | 0.87, 4.08 | 0.111 |  |  |  |  |  |
|  |  | Q4 | 1.76 | 0.65, 4.78 | 0.265 |  |  |  |  |  |
|  |  |  |  |  |  |  |  |  |  |  |
| BMI ≥ 30 kg/m^2^ | Yes | Q1 | Reference | |  | 0.063 | 1.01 | 0.99, 1.02 | 0.240 | 0.615 |
|  |  | Q2 | 2.49 | 0.52, 13.0 | 0.261 |  |  |  |  |  |
|  |  | Q3 | 1.97 | 0.44, 9.65 | 0.381 |  |  |  |  |  |
|  |  | Q4 | 2.30 | 0.52, 11.2 | 0.282 |  |  |  |  |  |
|  | No | Q1 | Reference | |  |  | 1.00 | 0.97, 1.03 | 0.856 |  |
|  |  | Q2 | 1.42 | 0.89, 2.28 | 0.144 |  |  |  |  |  |
|  |  | Q3 | 1.35 | 0.64, 2.81 | 0.419 |  |  |  |  |  |
|  |  | Q4 | - | - | - |  |  |  |  |  |
| Abdominal obesity | Yes | Q1 | Reference | |  | 0.379 | 0.98 | 0.95, 1.00 | 0.099 | 0.597 |
|  |  | Q2 | 1.63 | 0.92, 2.94 | 0.096 |  |  |  |  |  |
|  |  | Q3 | 1.59 | 0.88, 2.93 | 0.126 |  |  |  |  |  |
|  |  | Q4 | 1.37 | 0.65, 2.91 | 0.411 |  |  |  |  |  |
|  | No | Q1 | Reference | |  |  | 0.99 | 0.94, 1.04 | 0.647 |  |
|  |  | Q2 | 1.07 | 0.49, 2.35 | 0.861 |  |  |  |  |  |
|  |  | Q3 | 2.35 | 0.63, 8.57 | 0.197 |  |  |  |  |  |
|  |  | Q4 | 5.76 | 0.12, 283 | 0.345 |  |  |  |  |  |
|  |  |  |  |  |  |  |  |  |  |  |
| Arthritis | Yes | Q1 | Reference | |  | 0.512 | 0.98 | 0.95, 1.01 | 0.236 | 0.636 |
|  |  | Q2 | 1.50 | 0.86, 2.64 | 0.159 |  |  |  |  |  |
|  |  | Q3 | 1.74 | 0.92, 3.33 | 0.091 |  |  |  |  |  |
|  |  | Q4 | 1.68 | 0.71, 4.00 | 0.238 |  |  |  |  |  |
|  | No | Q1 | Reference | |  |  | 0.96 | 0.92, 1.01 | 0.091 |  |
|  |  | Q2 | 1.06 | 0.55, 2.04 | 0.858 |  |  |  |  |  |
|  |  | Q3 | 1.03 | 0.46, 2.30 | 0.949 |  |  |  |  |  |
|  |  | Q4 | 0.80 | 0.26, 2.39 | 0.686 |  |  |  |  |  |
|  | | | | | | | | | | |
| Coronary heart disease | Yes | Q1 | Reference | |  | 0.620 | 0.95 | 0.91, 1.00 | 0.043 | 0.165 |
|  |  | Q2 | 0.92 | 0.47, 1.80 | 0.799 |  |  |  |  |  |
|  |  | Q3 | 0.89 | 0.40, 2.01 | 0.786 |  |  |  |  |  |
|  |  | Q4 | 0.58 | 0.18, 1.87 | 0.367 |  |  |  |  |  |
|  | No | Q1 | Ref. | Ref. |  |  | 0.98 | 0.95, 1.02 | 0.318 |  |
|  |  | Q2 | 1.75 | 1.00, 3.09 | 0.053 |  |  |  |  |  |
|  |  | Q3 | 2.04 | 1.05, 4.00 | 0.037 |  |  |  |  |  |
|  |  | Q4 | 1.98 | 0.83, 4.74 | 0.125 |  |  |  |  |  |
|  |  |  |  |  |  |  |  |  |  |  |
| Heart attack | Yes | Q1 | Reference | |  | 0.324 | 0.95 | 0.91, 0.99 | 0.013 | 0.214 |
|  |  | Q2 | 1.04 | 0.54, 2.02 | 0.906 |  |  |  |  |  |
|  |  | Q3 | 0.77 | 0.35, 1.71 | 0.521 |  |  |  |  |  |
|  |  | Q4 | 0.64 | 0.21, 1.94 | 0.434 |  |  |  |  |  |
|  | No | Q1 | Reference | |  |  | 1.00 | 0.96, 1.03 | 0.908 |  |
|  |  | Q2 | 1.77 | 0.99, 3.18 | 0.055 |  |  |  |  |  |
|  |  | Q3 | 2.38 | 1.23, 4.67 | 0.011 |  |  |  |  |  |
|  |  | Q4 | 2.31 | 0.96, 5.61 | 0.064 |  |  |  |  |  |
|  |  |  |  |  |  |  |  |  |  |  |
| Cancer & Malignancy | Yes | Q1 | Reference | |  | 0.929 | 1.02 | 0.96, 1.09 | 0.551 | 0.515 |
|  |  | Q2 | 1.50 | 0.62, 3.64 | 0.371 |  |  |  |  |  |
|  |  | Q3 | 2.22 | 0.77, 6.57 | 0.144 |  |  |  |  |  |
|  |  | Q4 | 2.56 | 0.50, 13.4 | 0.258 |  |  |  |  |  |
|  | No | Q1 | Reference | |  |  |  |  |  |  |
|  |  | Q2 | 1.34 | 0.83, 2.19 | 0.232 |  | 0.96 | 0.94, 0.99 | 0.010 |  |
|  |  | Q3 | 1.39 | 0.79, 2.46 | 0.255 |  |  |  |  |  |
|  |  | Q4 | 1.18 | 0.56, 2.49 | 0.668 |  |  |  |  |  |
|  |  |  |  |  |  |  |  |  |  |  |
| Hypertension | Yes | Q1 | Reference | |  | 0.280 | 0.97 | 0.94, 1.00 | 0.032 | 0.219 |
|  |  | Q2 | 1.32 | 0.82, 2.12 | 0.259 |  |  |  |  |  |
|  |  | Q3 | 1.43 | 0.83, 2.48 | 0.201 |  |  |  |  |  |
|  |  | Q4 | 1.02 | 0.49, 2.14 | 0.955 |  |  |  |  |  |
|  | No | Q1 | Reference | |  |  | 1.01 | 0.95, 1.08 | 0.692 |  |
|  |  | Q2 | 1.08 | 0.38, 3.03 | 0.884 |  |  |  |  |  |
|  |  | Q3 | 1.28 | 0.35, 4.81 | 0.714 |  |  |  |  |  |
|  |  | Q4 | 4.10 | 0.63, 28.0 | 0.142 |  |  |  |  |  |
|  |  |  |  |  |  |  |  |  |  |  |
| Stroke | Yes | Q1 | Reference | |  | 0.212 | 0.96 | 0.90, 1.02 | 0.158 | 0.640 |
|  |  | Q2 | 1.07 | 0.42, 2.75 | 0.895 |  |  |  |  |  |
|  |  | Q3 | 2.11 | 0.67, 6.78 | 0.205 |  |  |  |  |  |
|  |  | Q4 | 0.83 | 0.16, 4.20 | 0.821 |  |  |  |  |  |
|  | No | Q1 | Reference | |  |  | 0.97 | 0.95, 1.00 | 0.073 |  |
|  |  | Q2 | 1.35 | 0.84, 2.18 | 0.223 |  |  |  |  |  |
|  |  | Q3 | 1.21 | 0.70, 2.12 | 0.498 |  |  |  |  |  |
|  |  | Q4 | 1.30 | 0.62, 2.73 | 0.494 |  |  |  |  |  |

GNRI grouping by Q1: GNRI ≤109.95, Q2: 109.95< GNRI ≤118.67, Q3: 118.67< GNRI ≤128.53, Q4: 128.53< GNRI.

Multivariate Cox proportional hazards model adjusted for age, gender, race, education, PIR, nutrients (protein and fiber intake), BMI, abdominal obesity, smoking, drinking, comorbidities (arthritis, coronary heart disease, angina, heart attack, stroke, liver condition, cancer & malignancy, hypertension), medications (hypertension, cholesterol), laboratory measurements (serum glucose, serum iron, serum creatinine).

Abbreviations: BMI, body mass index; CI, confidence interval; GNRI, geriatric nutrition risk index; HF, heart failure; OR, odds ratio; PIR, poverty income ratio.
